# Endoplasmic Reticulum Stress in Renal Cell Carcinoma

**DOI:** 10.3390/ijms24054914

**Published:** 2023-03-03

**Authors:** Marta Correia de Sousa, Etienne Delangre, Miranda Türkal, Michelangelo Foti, Monika Gjorgjieva

**Affiliations:** Department of Cell Physiology and Metabolism, Faculty of Medicine, University of Geneva, CH-1211 Geneva, Switzerland

**Keywords:** renal cell carcinoma (RCC), chronic kidney disease (CKD), endoplasmic reticulum (ER) stress

## Abstract

The endoplasmic reticulum is an organelle exerting crucial functions in protein production, metabolism homeostasis and cell signaling. Endoplasmic reticulum stress occurs when cells are damaged and the capacity of this organelle to perform its normal functions is reduced. Subsequently, specific signaling cascades, together forming the so-called unfolded protein response, are activated and deeply impact cell fate. In normal renal cells, these molecular pathways strive to either resolve cell injury or activate cell death, depending on the extent of cell damage. Therefore, the activation of the endoplasmic reticulum stress pathway was suggested as an interesting therapeutic strategy for pathologies such as cancer. However, renal cancer cells are known to hijack these stress mechanisms and exploit them to their advantage in order to promote their survival through rewiring of their metabolism, activation of oxidative stress responses, autophagy, inhibition of apoptosis and senescence. Recent data strongly suggest that a certain threshold of endoplasmic reticulum stress activation needs to be attained in cancer cells in order to shift endoplasmic reticulum stress responses from a pro-survival to a pro-apoptotic outcome. Several endoplasmic reticulum stress pharmacological modulators of interest for therapeutic purposes are already available, but only a handful were tested in the case of renal carcinoma, and their effects in an in vivo setting remain poorly known. This review discusses the relevance of endoplasmic reticulum stress activation or suppression in renal cancer cell progression and the therapeutic potential of targeting this cellular process for this cancer.

## 1. The Kidney and Renal Cell Carcinoma

The kidney carries out key physiological functions in the organism including blood filtration and pressure regulation, drug metabolism and glycemia control, as well as excretion of toxic metabolites. Tubular cells are the most abundant cell type of the kidney, and are major actors in the filtration/reabsorption processes and glycaemia control exerted by the kidney [1,2,3]. These cells are thus highly metabolically active and have high energetic requirements, usually satisfied by lipid β-oxidation but also through glycolysis in pathological conditions [3,4]. This variety of functions renders tubular cells particularly susceptible to stress-induced cell injury associated with drugs/metabolites toxicity and ischemic episodes, which with chronicity can lead to the development of renal cancer.

### 1.1. Renal Cell Carcinoma Epidemiology and Mutational Profiles

Renal cell carcinoma (RCC) mostly arises from tubular cells, although other kidney cell types were also suggested be at the origin of this cancer [5]. GLOBOCAN data reported over 400,000 new cases in 2020, accounting for 2% of all cancer diagnoses, with a higher prevalence in male patients [6]. Risk factors include smoking, obesity, diabetes, hypertension, chronic kidney disease and exposure to radiation and toxins such as trichloroethylene [6]. Other rare hereditary conditions, such as von Hippel-Lindau syndrome, Birt-Hogg-Dubé syndrome and Tuberous Sclerosis syndrome, can also contribute to the incidence of RCC [7]. The survival of the patients is strongly dependent on the stage of the disease at the time of diagnosis. The staging of the tumors (I–IV) is based on their size and invasiveness, with only 12% survival rate in the 5 years following diagnosis for patients with stage IV tumors [5,6].

RCC englobes a very heterogenous group of cancers in the kidney. The three major groups of RCC are clear cell RCC (ccRCC), papillary RCC (pRCC) and chromophobe RCC (chRCC), with ccRCC being the most frequent type (around 80% of all RCC) [8]. Histologically, ccRCC cells are characterized by a cytoplasm rich in lipids and glycogen, giving a clear cell aspect to this tumoral subtype [9]. These lipid and glycogen accumulations result from striking alterations of the cellular metabolism, as discussed below [8]. This cancer type originates from proximal tubular cells in the kidney. On the contrary, pRCC are tumors with smaller cells organized in a papillary architecture, with either basophilic (type I) or eosinophilic (type II) cytoplasm [9]. pRCC can also originate from proximal tubular cells, yet single-cell analysis suggested that this subgroup could arise from kidney collecting duct principal cells as well [10]. Finally, chRCC are characterized by large, pale cells with peri-nuclear halos and reticular cytoplasm, and they originate from distal convoluted tubules [9,11]. The mutation profile in RCC varies depending on the different types of tumors. Indeed, the most commonly mutated gene in ccRCC is, by far, the von Hippel-Lindau tumor suppressor gene (*VHL*) [12], found to be genetically altered in up to 60% of all ccRCC [13]. Other frequently mutated genes include Polybromo 1 (*PBRM1*), BRCA-associated protein 1 (*BAP1*) and SET Domain Containing 2 (*SETD2*) [14]. pRCC, on the other hand, frequently bears mutations in the hepatocyte growth factor receptor (*MET* proto-oncogene), *SETD2* and Moesin-Ezrin-Radixin Like Tumor Suppressor (*NF2*), while chRCC tumors are characterized by *TP53* and Phosphatase and tensin homolog (*PTEN*) mutations [14].

### 1.2. Metabolic Reprogramming in Renal Cell Carcinoma

While metabolic alterations in pRCC and chRCC are still poorly characterized, a deep reprogramming of the energetic metabolism was highlighted in ccRCC by several studies [8,15]. Consistent with the high levels of lactate found in the urine of patients [16], ccRCC undergo metabolic switches that increase glycolysis and lactate fermentation to fuel cell proliferation [15,17]. Glucose uptake through the GLUT1 glucose transporter and glycogen accumulation are increased in ccRCC [15,17,18,19,20], and glycolytic intermediates partition to feed both the pentose phosphate pathway (PPP) and the TCA cycle and one carbon metabolism [15,17]. The increased stimulation of the PPP allows the nucleotide synthesis required for cell proliferation, but this metabolic rewiring also attenuates mitochondrial activity and respiration, thus preserving lipids and cholesterol for membrane production and signalization while protecting the cells from oxidative stress by decreasing mitochondrial reactive oxygen species (ROS) production [15,17,21]. This classical Warburg effect further increases with the severe clinical stage of ccRCC [22,23]. The expression of glycolytic enzymes, e.g., glucose-6-phosphate isomerase (GPI), GLUT1 and MCT1, also increases with the different ccRCC stages and represents the independent prognostics marker for this cancer type [19,24]. Accordingly, glycolytic gene-related signatures in RCC patient cohorts correlate with the prognosis and therapeutic responses of the patients [25,26].

As described later on in detail, loss of the tumor suppressor *VHL* in ccRCC triggers the expression of hypoxia-induced factor 1 (HIF1), a glycolytic transcription factor [27]. However, HIF1 activity is not restricted to glycolysis promotion; other metabolic pathways are also under the HIF1 control. Integrated multi-omics analysis of human ccRCC samples revealed that (i) NDUFA4L2 targeted by HIF1 triggers mitochondrial dysfunction and blockage of mitochondrial respiration through inhibition of the Complex I of the respiration chain [28]; (ii) HIF1 interactor MUC1 regulates glycogen degradation, glycolysis, PPP, TCA cycle in RCC [29]; and (iii) HIF1-mediated transcription of PFKFB4 promotes PPP activation in RCC [30,31]. Further supporting the importance of the PPP for carcinogenesis, overexpression of glucose-6-phosphate dehydrogenase (G6PDH), a rate-limiting enzyme in the PPP, was shown to favor cell survival and to protect RCC cells from oxidative stress, while its inhibition by 6-aminonicotinamide resulted in decreased NADPH levels and increased ROS concentration in primary renal tumor cells [31].

Consistent with the impairment of mitochondrial respiration and increased PPP in ccRCC, the lipid metabolism is also strongly deregulated in this cancer type. Aberrant accumulation of lipids occurs in ccRCC cells, thus contributing to the clear aspect of these cancer cells in histology. Lipidomic analysis of human ccRCC confirmed an extensive accumulation of lipids in these cancer cells, in particular ether phospholipids, cholesterol esters, and triacylglycerols [32]. Supporting an aberrant accumulation of lipids in ccRCC cells, analysis of early stage human ccRCC revealed an increased expression of the lipid transporter CD36, the lipid synthesis enzymes SCD1 and ELOVL2, and the structural component of lipid droplets PLIN2, as well as downregulation of ANXA3, a negative regulator of lipid accumulation [21,33].

### 1.3. Therapeutic Options for Renal Cell Carcinoma

Treatment options for RCC have varied greatly over the years. Almost two decades ago, the most exploited options were IL-2- and IFNα-based therapies, despite the underlying toxicity of these treatments [34]. Advancements in drug development then led to the extensive usage of tyrosine kinase inhibitors (TKI), such as Sunitinib, Sorafenib, Axitinib and Cabozantinib [35]. Inhibitors of the mTOR pathway, such as Everolimus and Temsirolimus, were also considered, as this pathway is frequently upregulated in RCC [4]. Moreover, targeting of the VEGF angiogenic pathway was also exploited using Sunitinib or Bevacizumab and in further combination with PD-1 inhibitors such as Pembrolizumab [36]. Indeed, immune checkpoint inhibitors (ICI) are increasingly considered as treatment for RCC, as well as the combination of ICI with TKI. Therapeutic approaches based on ICI are highly relevant, since RCC are among the most immune-infiltrated tumors [37,38]. Interestingly, evidence is emerging suggesting that the activation of specific metabolic pathways is tightly associated with inflammatory signatures and angiogenesis [39,40]. In this regard, in silico analyses suggested that high metabolic activity in ccRCC tumors can suppress immune infiltration and that both metabolic and immune status of the tumors could be used for prognosis [41]. In agreement with this concept and the fact that the tumor microenvironment heavily affect responses to systemic therapy [42], patients with RCC characterized by high inflammation and low metabolic activity are the ones that benefit mostly from immunotherapy [41].

The most commonly used TKI show conflicting results regarding the beneficial effect on patients, as demonstrated in different RCC patient cohort studies (e.g., study ASSURE vs. S-TRAC for Sunitinib and Sorafenib [43]), indicating that the underlying molecular mechanisms involved in RCC are more complex and likely need combined therapies. Nephrectomy can also be envisaged as an option, particularly in advanced cases of RCC. However, very few patients are eligible for these procedures, which are limited by the location and accessibility of the tumor, the associated comorbidities and the extent of the symptoms [44]. Finally, in patients with metastatic RCC, radiotherapy can also be employed, but the survival of the patients is poorly improved [45]. Therefore, novel therapeutic options are needed to optimize the treatment for RCC.

As mentioned, current therapies in RCC, in particular TKI, exhibit poor efficiency and lack in durable results. For example, more than one quarter of RCC patients undergoing Sunitinib or Sorafenib treatment were reported to be primary refractory to this therapeutic approach [46]. This resistance to the treatment can be due to the specific mutational profile of the tumor and the genetic background of the patient, as well as different redundant pro-angiogenic mechanisms that allow cancer cells to bypass the pathways targeted by the TKI and insure their survival [46]. One other major factor that can facilitate renal cancer cell survival with TKI-based therapies is the induction of endoplasmic reticulum (ER) stress, which in turn activates pro-inflammatory/pro-survival molecular mechanisms that foster RCC progression. Indeed, both Sunitinib and Sorafenib have been demonstrated to induce ER stress responses in RCC cells [47,48].

In the following sections, we discuss how ER stress signaling promotes renal cancer cells survival, and how these molecular pathways could be either hyperactivated or suppressed as two different therapeutic strategies against RCC.

## 2. Endoplasmic Reticulum Stress Signalling in Pro-Survival and Pro-Apoptotic Mechanisms

The ER is an organelle that exerts various vital functions in the cell, such as protein folding, lipid synthesis, regulation of carbohydrate metabolism and Ca^2+^ homeostasis [49]. The ER compartment thus represents an important cellular site at the crossroad of a plethora of cellular processes regulating cell homeostasis. Alterations of ER functions drive cellular stresses that affect the structural and functional integrity of the ER and trigger signaling responses from this organelle known as the unfolded protein response (UPR). While UPR activation is a molecular process aiming at restoring homeostasis in the cell following an insult, its prolonged activation can lead to programmed cell death. A multitude of different stress factors can affect ER functions and lead to the activation of the UPR. These include, in particular, (i) metabolic alterations such as hyperglycemia, hyperlipidemia and nutrient availability, (ii) abnormalities in the Ca^2+^-dependent signaling, (iii) mutational changes that favor a constitutive activation of the UPR (frequently observed in cancer), (iv) damage induced by reactive oxygen species (ROS), (v) heat shock, and (vi) toxin/drug exposure [50].

**Figure 1:**
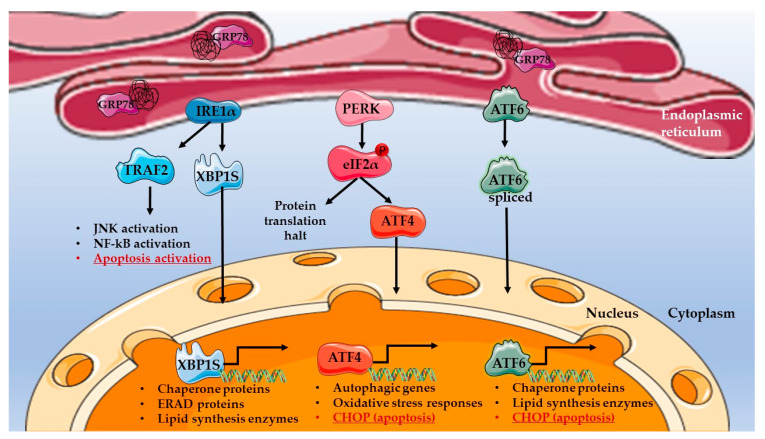
Unfolded protein response (UPR) pathway in ER stress conditions. The three different UPR axes are represented as follows: IRE1α-mediated pathways are in blue, PERK-mediated pathways are in red and ATF6 in green. Pro-apoptotic UPR responses are underlined in red. The UPR sensor GRP78 is represented in violet, bound to misfolded proteins (in black) in the ER lumen. The illustration was designed using images from the Servier Medical art database (smart.servier.com, accessed on 26 December 2022).

ER stress activates three main UPR signaling axes, which are under the control of the inositol requiring enzyme 1 (IRE1α), the PKR-like ER kinase (PERK) and activating transcription factor 6 (ATF6), respectively (Figure 1) [51]. Each of these signaling axes exerts a particular role in re-establishing normal homeostasis following cell injuries. IRE1α, PERK and ATF6 are ER-transmembrane proteins, and their activation is dependent on sensory proteins, among which the best-characterized is glucose-related protein 78 (GRP78, also called BiP) [52]. GRP78 is a resident protein in the ER lumen that binds to IRE1α, PERK and ATF6 in the absence of ER stress to prevent the UPR. Upon ER stress, GRP78 dissociates from the ER-lumen domains of IRE1α, PERK and ATF6, thus allowing their activation. GRP78 dissociation from the UPR drivers is triggered by the higher affinity of GRP78 for misfolded proteins, which accumulate in the ER in stress conditions. Other ER stress sensors, e.g., sarco-ER calcium-ATPase (SERCA) and ER-associated calcium sensors stromal interacting molecule (STIM), can also drive UPR activation, but through indirect mechanisms modulating Ca^2+^ levels in the ER [52].

Dissociation of GRP78 from IRE1α allows autophosphorylation and conformational changes of IRE1α, conferring to it cytoplasmic nuclease activity towards the mRNA of the XBP1 transcription factor, which is spliced into the XBP1S active mRNA variant. The XBP1S protein then migrates to the nucleus, where it activates the transcription of chaperone proteins, as well as other factors in the ER-associated protein degradation (ERAD) pathway, to restore proteostasis (Figure 1) [53]. Increased expression of chaperone proteins alleviates ER stress by preventing further accumulation of misfolded proteins and/or inducing their degradation. The XBP1S transcription factor was also shown to regulate metabolism, in particular through the activation of lipogenesis by stimulating the transcription of acetyl-CoA carboxylase 2 (*ACC2*), stearoyl-CoA desaturase 1 (*SCD1*) and diacylglycerol acyltransferase 2 (*DGAT2*) [53]. Therefore, while metabolic alterations themselves induce ER stress, this mechanism can further contribute to these abnormalities by stimulating lipid synthesis in a vicious cycle. 

The second axis of the UPR mediated through PERK is responsible for the decrease in protein synthesis under ER stress. PERK is activated upon GRP78 dissociation and phosphorylates the eukaryotic translation initiation factor 2α (eIF2α), halting global protein translation, yet the production of specific proteins, such as activating transcription factor 4 (ATF4), is induced. ATF4 in turn triggers the transcription of oxidative stress response proteins and autophagy-related proteins (Figure 1).

Finally, the mediator of the third axis, the ATF6 transcription factor, is activated by cleavage upon GRP78 dissociation. Active ATF6 then translocates into the nucleus and promotes the transcription of genes encoding chaperone proteins and enzymes of the lipid metabolism, as well as proteins involved in UPR mediation, such as XBP1S and GRP78 (Figure 1) [54]. In this way, ATF6 tends to alleviate protein burden in the ER, but is also responsible for metabolic changes in the cell, reducing cell stress.

As mentioned, the UPR initially aims to restore the normal functioning of the ER through the activation of chaperone proteins by decreasing protein synthesis, regulating the lipid metabolism and activating other stress responses, such as autophagy and antioxidant activity [55]. Together these responses mediate the pro-survival phase of the UPR (also called adaptive phase of the UPR). Nevertheless, if the initial cause triggering the ER stress is not resolved and the stress stimuli persist, the continuous activation of the UPR leads to cell death (pro-apoptotic response of the UPR). UPR-dependent apoptosis can be conveyed via different pathways, including: (i) ATF4- and ATF6-mediated activation of CCAAT/enhancer binding protein homologous protein (CHOP) transcription, which is the main pro-apoptotic actor of ER stress; (ii) IRE1α-induced activation of tumor necrosis factor α (TNFα) receptor-associated factor 2 (TRAF2), which leads to JNK activation and apoptosis; or (iii) IRE1α-induced activation of Caspases [56,57]. 

The ability of the UPR to trigger either pro-survival or pro-apoptotic mechanisms, when the cellular damage is deemed irreversible, is very attractive for therapeutic purposes in diseases such as cancer and needs attention when considering personalized medicine approaches. To exploit these molecular pathways as therapeutic targets, several critical factors in ER stress signaling need to be better understood. First, it is important to precisely define the threshold of cell damages above which ER stress becomes irreversible and triggers the activation of the pro-apoptotic machinery. This limit, from which the UPR induces a switch from a pro-survival to a pro-apoptotic response, still remains obscure and is thought to be highly variable between different cell types, tissues and/or organs. Furthermore, the main cellular actors governing this switch need to be identified and characterized, as well as whether the three signaling axes of the UPR are equally important in the switch from anti- to pro-apoptotic mechanisms induction. In this regard, the E2F1 transcription factor was suggested to act as a molecular switch indispensable for ER stress-mediated activation of apoptosis [58]. Indeed, the downregulation of E2F1 in vitro under ER stress conditions leads to the upregulation of the pro-apoptotic factors Noxa and Puma, which are required for ER stress-induced apoptosis [58]. Nevertheless, such mechanism still needs to be experimentally confirmed in animal models and in humans. 

## 3. ER Stress Signaling in Renal Cell Carcinoma

**Figure 2:**
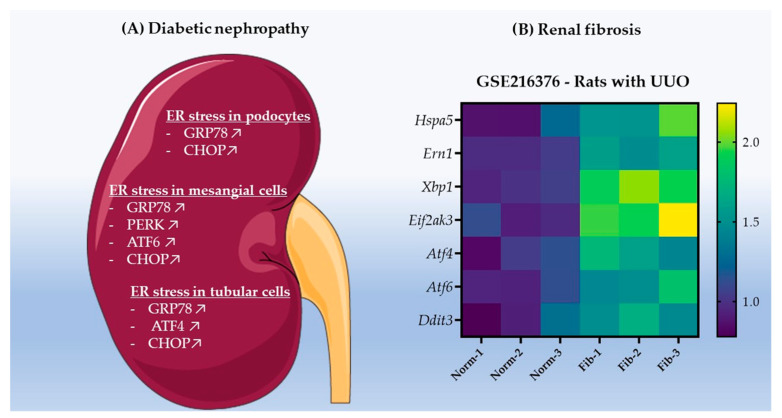
ER stress activation in diabetic nephropathy and renal fibrosis. (**A**) Schematic representation of the different UPR mediators upregulated in podocytes [79,80], mesangial cells [81,82,83] and tubular cells [84,85,86] in diabetic nephropathy. The illustration was designed using images from the Servier Medical art database (smart.servier.com). (**B**) Heatmap representation of the expression of *Hspa5* (encoding GRP78), *Ern1* (encoding IRE1α), *Xbp1*, *Eif2ak3* (encoding PERK), *Atf4*, *Atf6* and *Ddit3* (encoding CHOP) in the kidneys of normal adult rats that underwent sham surgery (*n* = 3; Norm) and adult rats that underwent unilateral ureter obstruction (UUO) (*n* = 3; Fib) in order to induce renal fibrosis development. The data were acquired from the GEOdataset GSE216376 (https://www.ncbi.nlm.nih.gov/geo/query/acc.cgi?acc=GSE216376 accessed on 26 December 2022–expression profiling by high throughput sequencing). Graphical representation of the fold-change to control group was designed with the GraphPad Prism 9 software.

Chronic kidney diseases (CKD) are a favorable ground for RCC development, and patients suffering from these diseases are at higher risk of developing renal cancer, in comparison to the general population [59,60]. Metabolic abnormalities, ischemia, abnormal redox homeostasis, inflammation and fibrosis are all drivers of carcinogenesis, as well as major hallmarks of CKD [61,62,63]. As illustrated in Figure 2, in the case of diabetic nephropathy (DN) [64,65,66] and renal fibrosis [67], which occurs in almost all CKD [68], ER stress and activation of the UPR develop in both acute and chronic injuries in the kidney [69], including ischemic and nephrotoxic acute kidney injury [70], alcoholic nephropathy [71], autosomal dominant tubulointerstitial kidney disease [72], autosomal dominant polycystic kidney disease [73], membranous nephropathy [74,75], Fabry disease [76] and lupus nephritis [77]. As further described in this section, ER stress often occurs in tubular cells from which most RCC originate, e.g., in DN [78], thus not only contributing to aggravating CKDs, but also providing a further priming for RCC development.

### 3.1. ER Stress Promotes Renal Cell Carcinoma Development in Chronic Kidney Disease

The underlying pathology driving CKD determines the type of RCC that the patient is most likely to develop [60]. For example, in a CKD such as DN, metabolic alterations induce oxidative stress, renin-angiotensin-aldosterone system (RAAS) activation and immunological changes in the kidney that promote cancer induction and progression [87]. Oxidative stress can induce DNA damage in the cell, facilitating tumoral transformation. Moreover, chronic exposure of proximal tubular cells to oxidative stress leads to the acquisition of stem cell-like features/markers facilitating tumoral transformation, as observed in HK-2 normal kidney tubular cells [88]. The RAAS is known to induce renal fibrosis and thus create a deleterious microenvironment (e.g., hypoxia, hyper-proliferative state), priming the kidney for renal carcinogenesis. High circulating glucose and lipids also promote cell proliferation. Hyperglycemia was suggested to foster DN-associated RCC development [89] by (i) hyperactivating glycolysis (reported in transcriptomic/metabolomics analysis of diabetic mouse kidneys [90]) and (ii) activating the AKT/mTOR and the insulin growth factor (IGF) proliferative signaling pathways, both shown to be activated in DN in mouse models of diabetes [91,92]. Hyperlipidemia promotes lipid uptake in renal cells via the CD36 transporter, resulting in ectopic accumulation of lipids in cytoplasmic droplets of kidney cells, as observed in immortalized mouse kidney cells stimulated with lipids in vitro, as well as in human diabetic kidney biopsies [93,94]. While lipid droplet accumulation per se seems to not be deleterious for tubular cells, overcoming the lipid storage capacities of these cells becomes toxic (lipotoxicity) and was suggested to stimulate cancer initiation; nevertheless, concrete experimental data are still missing [94]. Intracellular free fatty acids and their metabolites in renal cells can indeed interact with DNA, RNA, proteins and organelles and thereby affect their normal functioning, entailing genetic instability that fosters cancer development, as previously suggested in vivo in mouse models with renal metabolic alterations similar to DN (K.G6pc-/-mouse model) in which renal neoplasia development was noted [95,96,97]. Finally, inflammation associated with DN might also contribute to renal carcinogenesis, as is the case for many other cancers, but this remains to be clearly demonstrated [62,98,99]. Inflammatory mediators mainly produced by immune cells that infiltrate the kidney, but also renal cells in the kidney cortex, e.g., IL-6, TNF-α, IL-1β, and related signaling pathways such as JNK and NF-kB, were reported to significantly contribute to the development and progression of DN in mouse or rat streptozotocin-induced diabetic models of nephropathy [100,101,102], as well as major cancer hallmarks such as sustained proliferation, angiogenesis, immune escape and metastasis, which could facilitate renal cancer development with CKD [103,104].

All these metabolic alterations and inflammatory processes occurring in CKD and priming cells for carcinogenesis are tightly linked to ER stress, but whether ER stress is a cause or an effect of these alterations remains unclear. For example, tunicamycin-induced ER stress in non-cancerous renal epithelial HEK-293 cells induces lipotoxicity by increasing the intracellular content of long-chain ceramides and polyunsaturated fatty acids, which damage cells and trigger apoptosis [105]. On the other hand, in non-cancerous kidney tubular HK-2 cells, the decreased expression of *VHL*, a major tumor suppressor lost in kidney cancer, induces ER stress, as supported by GRP78, IRE1α/XBP1, eIF2α, JNK and NF-kB activation, as well as upregulation of NF-kB target genes (TNFα and Il-1β) [106]. *VHL* loss also resulted in an increased recruitment of macrophages and inflammation in a IRE1α-dependent manner in the same study [106]. This observation was reported in an in vitro setting in which the HK-2 cells with or without *VHL* expression were seeded in a Boyden chamber, allowing them to recruit or not recruit RAW264.7 macrophages through a porous membrane, respectively [106]. These findings suggest that the ER stress and UPR responses induced by the loss of *VHL* in pre-cancerous stages of kidney cells could represent an early event driving cell transformation and induction of RCC. 

The UPR, through activation of IRE1α, PERK and ATF6, could also contribute to maintaining an inflammatory environment, promoting carcinogenesis by stimulating the production and secretion of inflammatory mediators or modulating immune cell infiltration [107,108]. For example, the IRE1α axis of the UPR was shown to drive acute-to-chronic kidney disease transition in tubular cells by activating JNK signaling and by increasing IL-6 and MCP1 production and secretion in an in vivo model of renal ischemia/reperfusion [109]. PERK was further reported to upregulate the JAK2/STAT3 inflammation pathway in normal rat kidney NRK-52E cells [110]. Although the molecular mechanisms linking ER stress and inflammation remain to be deeply investigated, it is clear that a sustained inflammatory environment in the kidney evoked by ER stress signaling is likely an important factor priming the kidney for RCC development.

ER stress can modulate the autophagic capacity of renal cells [111]. Tunicamycin injection in mice to induce ER stress resulted in increased autophagy and apoptosis in the tubular cells of the kidneys through activation of PERK/eIF2α-dependent UPR, thus promoting chronic kidney injury [112]. Autophagy suppression in proximal tubular HK-2 cells in vitro, in turn, potentiated the activation of ER stress (as observed through an increase in GRP78 and eIF2α phosphorylation), thus suggesting an intricate relationship between these two cellular processes in kidney diseases. The authors suggest that autophagy activated in these conditions possibly provides a negative feedback regulation aiming to resolve the ER stress. Importantly, autophagy represents an important mechanism involved in the initiation and progression of RCC by allowing cells to recycle their intracellular materials/organelles to promote proliferation (as observed, for example, in vitro in Caki RCC cells, in which p53 is degraded in autophagic vesicles, favoring rapid proliferation of renal cancer cells [113,114]). 

Finally, ER stress leads to structural alterations of the ER cisternae, which may in turn affect the functions of other organelles in the cell [115]. For example, ER interactions with mitochondria and/or the plasma membrane through specific contact sites (mitochondria-associated endoplasmic reticulum membranes or MAMs) can be severely impaired upon ER stress [116,117]. The functional integrity of MAMs are indeed required for a normal ER or mitochondrial Ca^2+^ homeostasis, mitochondrial ROS production, fusion and lipid transfer, as well as for the cell metabolic homeostasis and processes such as apoptosis [118]. However, how the UPR and/or associated ultrastructural changes in the ER affect MAMs’ integrity, ER interactions with other organelles and their functions is still unclear in the context of RCC or kidney disease in general [119]. Only a few studies indicated that in an in vivo model of streptozotocin-induced DN, tubular cells and podocytes undergo a loss of MAMs integrity associated with an induction of apoptosis and renal injury [120,121]. Of note, studies with primary human ccRCC samples and retrospective analyses of ccRCC patients’ cohorts uncover significant mitochondrial dysfunctions and a decreased capacity for mitochondrial oxidation in this cancer subtype [122,123]. Further studies are now required to explore in more detail the pathological relevance of UPR-independent ER stress-associated cellular defects in the different subtypes of RCC. 

### 3.2. Mutations Dictate Metabolic-Dependent Activation of ER Stress in Renal Cell Carcinoma

As previously mentioned, metabolic reprogramming of renal cancer cells [124] is in part dependent on the type of mutations driving carcinogenesis [125]. For example, ccRCC in humans are characterized by aerobic glycolysis and pseudohypoxia, along with activation of the pentose phosphate pathway and a decreased oxidative phosphorylation, typical of a Warburg-like reprogramming [126]. These metabolic features are consistent with the recurrent loss of the tumor suppressor *VHL* in this cancer type. Indeed, *VHL* ubiquitinates and targets HIF1 for degradation, and loss of this tumor suppressor leads to HIF1 accumulation and activation of hypoxia responsive factors, glycolysis and glucose uptake in renal tubular cells [127]. *VHL* mutations are further associated with an upregulation of the pro-angiogenic vascular endothelial growth factor (VEGF) and mammalian target of rapamycin (mTOR) pathway, a major regulator of cell proliferation, energy metabolism and autophagy [127]. pRCC, like ccRCC, is also characterized by mTOR overactivation and loss of oxidative phosphorylation capacities; however, this cancer subtype is more dependent on glutamine consumption than glucose consumption in humans [128,129]. One of the most frequently mutated proto-oncogenes in pRCC, *MET*, can activate the PI3K-AKT-mTOR and LKB1-AMPK-mTOR nutrient-sensing pathway, which facilitates growth of the tumor [125]. Finally, loss of the tumor suppressor *PTEN,* often reported in human chRCC, can also re-wire the metabolism of RCC through activation of the PI3K/AKT pathway, thus fostering growth and progression of tumors [130,131]. However, sequencing of the mitochondrial DNA in this cancer subtype indicated that the mitochondrial oxidative phosphorylation pathway is not attenuated, in contrast to ccRCC [132]. Finally, the tumor suppressor *TP53* can be mutated in human chRCC, pRCC and ccRCC, thereby affecting not only the cell cycle and DNA repair but also the glucose metabolism by promoting a Warburg effect [133].

**Figure 3:**
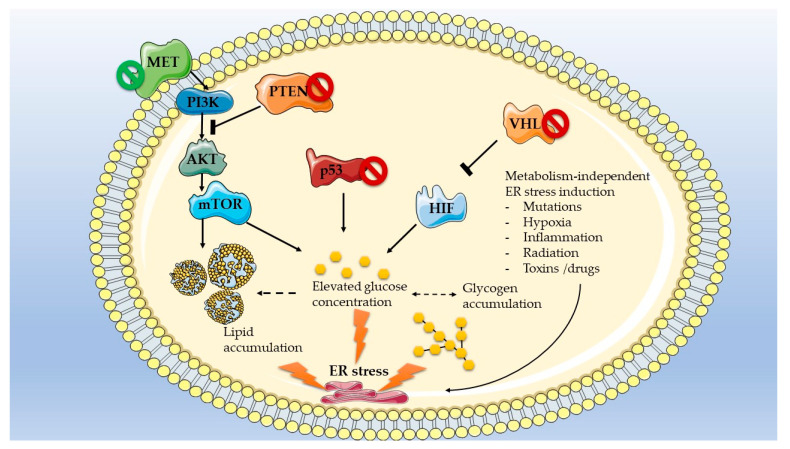
Frequent mutations in RCC and their impact on metabolism and metabolism-mediated ER stress activation. Tumor suppressors are represented in orange/red, pro-oncogenic factors are represented in green/blue. Proteins coded by frequently mutated genes are marked with the symbol Ø (green Ø indicates a mutation that increases gene expression/activity; red Ø indicates a loss of expression/activity). Graphical representation was designed using images from the Servier medical art database (smart.servier.com, accessed on 26 December 2022).

All the mutation-dependent metabolic alterations described for ccRCC, pRCC and chRCC are beneficial for the growth and progression of these cancers but also trigger severe stress responses, in particular from the ER. Ectopic accumulation of fat-containing droplets in ccRCC can indeed induce lipotoxicity and damage intracellular organelles, DNA, RNA and proteins, thereby activating the ER stress pathways in an attempt to restore homeostasis [134]. In addition, increased intracellular glucose levels exacerbate lipid-induced ER stress through the activation of lipogenesis and glucotoxicity [92]. Accordingly, lessons from diabetic nephropathy models have highlighted the importance of hyperglycemia and dyslipidemia in the activation of ER stress and the pathological outcome of the UPR on kidney disease [65]. Finally, abnormal activation of the mTOR pathway, which frequently occurs in ccRCC and pRCC, was also shown to activate ER stress responses in the kidney, as seen in podocytes in vitro [91]. The impact of the most frequent RCC mutations on metabolism and metabolism-mediated ER stress activation is summarized in Figure 3. It is crucial to stress that while ER stress responses are mostly induced by drastic metabolic alterations, other factors, such as exposure to drugs and toxins can also activate UPR in kidney cells.

Importantly, the activation of the UPR pathway can, in turn, regulate distinct metabolic activities, including lipid [135] and glucose metabolism [136]. This is exemplified by the overexpression of ATF6 in human tubular HK2 cells, which leads to a decrease in fatty acid oxidation, as well as the accumulation of lipids in these cells, causing mitochondrial dysfunction and apoptosis [137]. Therefore, while mutation-driven metabolic reprogramming can lead to ER stress activation, the latter can also modulate the metabolic activity of the tumor.

**Figure 4:**
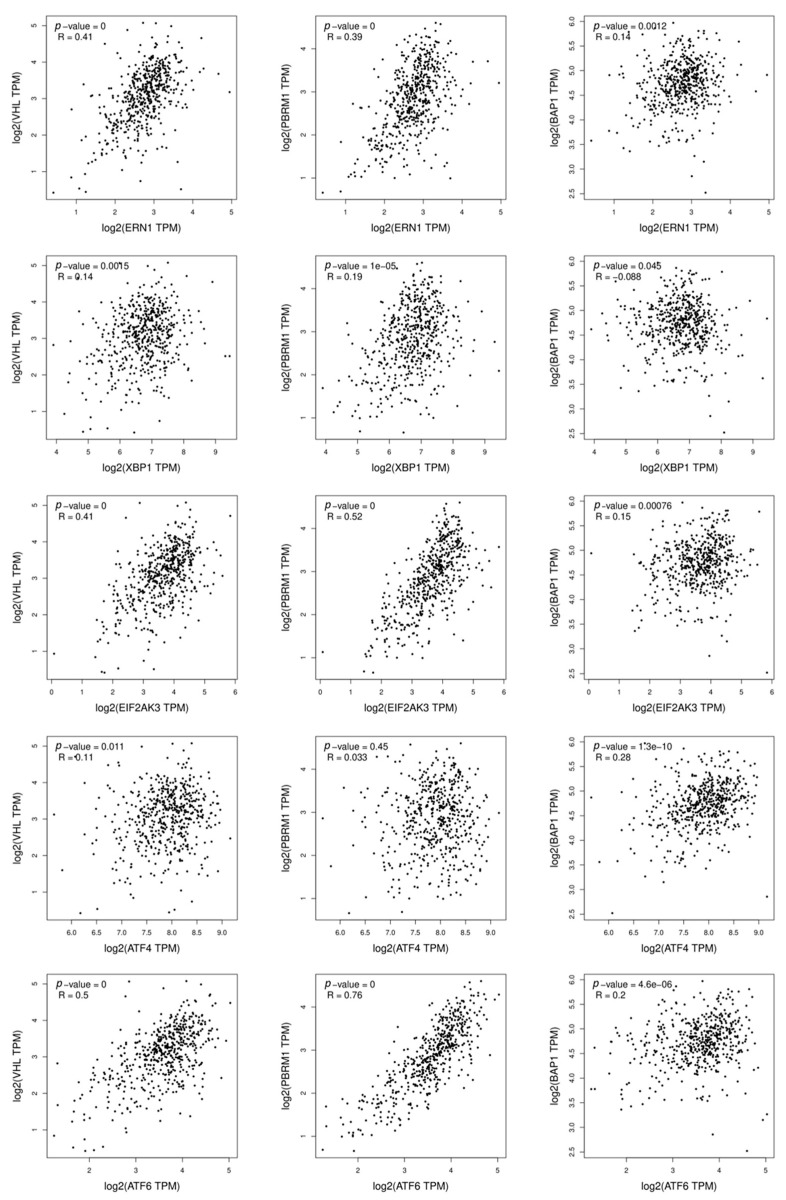
Correlation of ER stress markers and most commonly mutated genes in human ccRCC. Correlation of the expression of ER stress markers (*ERN1* (IRE1α), *XBP1*, *EIF2AK3* (PERK), *ATF4*, *ATF6*) versus the expression of most commonly mutated genes (*VHL*, *PBRM1* and *BAP1*) in human ccRCC form the TCGA-KIRC cohort (only tumoral tissues). The results shown here are in whole based upon data generated by the TCGA Research Network: https://www.cancer.gov/tcga accessed on 26 December 2022. Data were acquired and significance was assessed via the Gepia 2 cancer database (http://gepia2.cancer-pku.cn, accessed on 26 December 2022). The correlation coefficient used is Pearson. All of the correlations were statistically significant; however, the correlation was considered relevant only when the correlation coefficient R > 0.4.

Finally, expression of the three most frequently lost tumor suppressors (*VHL*, *PBRM1* and *BAP1*) in ccRCC do not correlate in the same manner with the expression of the different UPR mediators. Indeed, analyses of the TCGA-KIRC cohort of ccRCC patients via the Gepia2 cancer database (RCC biopsies of the patients) showed that *VHL* and *PBRM1* expressions correlate with *ERN1*, *EIF2AK3* and *ATF6*, whereas *BAP1* does not correlate with any of the ER stress mediators (Figure 4). These correlative data suggest that some of the most frequently mutated genes in RCC (*VHL* and *PBRM1*) might be able to regulate the activation status of the UPR, however, this activation does not apply to the three branches but rather a branch-specific mediation of the UPR. This would imply that the outcome of the UPR activation might be different depending on the mutation that is present in the RCC, thus modulating the type of ER stress signaling. Further studies are now required to evaluate in depth this hypothesis for its relevance to therapeutic strategies based on targeting of ER stress signaling, as discussed later in this review. 

### 3.3. UPR Gene Mutations in Renal Cell Carcinoma

As described in the previous section, metabolic alterations driven by specific mutations can activate ER stress in RCC. Nevertheless, the genes coding for the different proteins involved in ER stress signaling can also be mutated and lead to constitutive activation of the UPR, promoting cancer development. For example, the gene coding for IRE1α (*ERN1*) was shown to be frequently mutated (increased copy number) and to contribute to breast cancer malignancy, with a specific subtype of breast cancer (luminal B breast cancer) showing the strongest *ERN1* gene gain/amplification frequency, over 68%, as observed through in silico assessment of the Pan-Cancer Atlas of The Cancer Genome Atlas (TCGA) [138]. However, in RCC, activating mutations of UPR mediators are uncommon. Indeed, analyses of five different cohorts of ccRCC (Kidney Renal Clear Cell Carcinoma (TCGA, PanCancer Atlas), Clear Cell Renal Cell Carcinoma (DFCI, Science 2019), Kidney Renal Clear Cell Carcinoma (BGI, Nat Genet 2012), Kidney Renal Clear Cell Carcinoma (IRC, Nat Genet 2014), Renal Clear Cell Carcinoma (UTokyo, Nat Genet 2013), combining together 761 patients and using the cBioportal (www.cbioportal.org, accessed 24 October 2022) database, indicated that the frequency of mutations in ER stress mediators is extremely low in RCC. Indeed, *HSPA5* (GRP78) was mutated in 2/761 patients (missense mutations), *ERN1* (IRE1α) in 2/761 patients (missense mutation and frame shift deletion), *ATF4* in 3/761 patients (2 missense and 1 in frame deletion), *ATF6* in 2/761 patient (in frame deletion) and *XBP1*, *EIF2AK3* (PERK) and *DDIT3* (CHOP) were not mutated in any of the patients. It is therefore clear that the frequent activation of ER stress responses in RCC cannot be attributed to mutational changes in genes encoding the UPR mediators. 

## 4. Targeting Endoplasmic Reticulum Stress Pathways in Renal Cancer

Based on normal cell physiology, induction of ER stress should be beneficial against pathologies such as cancer, since it should induce the death of transformed cells. However, while a normal cell would either resolve ER stress or undergo apoptosis, the profound changes characterizing transformed cells can lead to different outcomes of ER stress. Indeed, prolonged activation of the UPR does not always induce apoptosis in cancer cells, one of the main characteristics of which is precisely resistance to cell death. Instead, mild and prolonged UPR activation leads to mutational and metabolic adaptations of the transformed cell, which in turn favor its growth, metabolic status and proliferation/migration capacities [139]. In this regard, several UPR mediators were reported to favor cancer cell survival and proliferation. For example, ATF4 was shown to be critical for the survival of fibrosarcoma and colorectal adenocarcinoma cells under nutrient deprivation in vitro [140] and could also promote neoplastic transformation of mouse primary embryo fibroblasts by inhibiting senescence factors [141]. Overexpression of GRP78 was found to exert an anti-apoptotic role through the blockage of apoptotic mediators such as caspase-7 or BIK in breast cancer in vitro [142,143]. PERK was reported to induce resistance to cell death and chemotherapy and to confer NRF2-dependent protection to colon cancer HT29 cells against oxidative stress [144]. ATF6 was shown to sustain the expression of oncogenes such as BRCA1 or CIP2A (in vitro data in human colon cancer cell lines CaCO2, and SW480), as well as prevent DNA damage and to improve cancer cell viability, as observed in RKO and HCT116 colon cancer cell lines [145,146].

**Figure 5:**
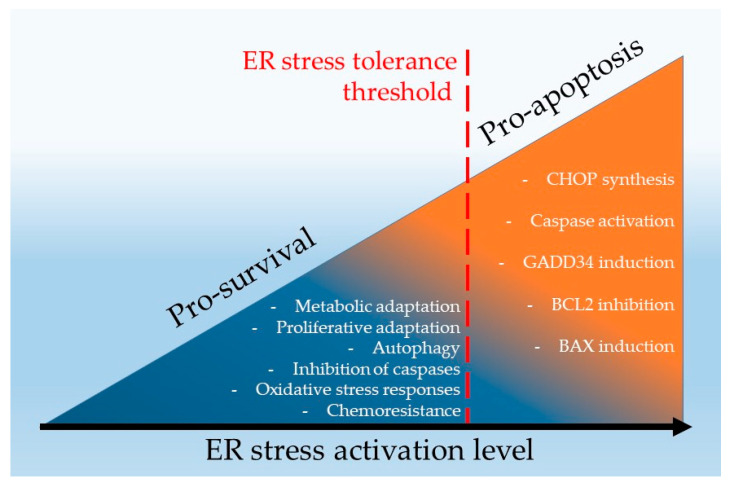
Dichotomous function of ER stress in renal cancer cells.

These observations suggest that the induction of ER stress can lead to cancer cell death only in certain conditions allowing it to surpass the resistance threshold of the tumoral cells, also called ER stress tolerance or ERST. ERST can be highly dependent on the mutations, tumor microenvironment and the progression stage. (Figure 5). Therefore, two therapeutic strategies targeting ER stress to eliminate cancer cells in RCC can be considered. On one hand, hyperactivation of ER stress to tip over the ERST and induce cell death appears as an interesting approach [139]; the second option that could be envisaged is to inhibit ER stress in RCC cells in order to facilitate cell death mediated by other therapeutics, such as TKI. Both approaches are discussed in the following sections.

### 4.1. Pharmacological Hyperactivation of ER Stress as a Strategy to Overcome the ERST and to Induce RCC Cell Death

Various studies analyzed combinatory treatments in pre-clinical settings, aiming to increase ER stress in order to induce cell death as a therapeutic option in RCC. The combined usage of GZ17-6.02 (curcumin, harmine and isovanillin) with axitinib, a tyrosine kinases inhibitor (TKI), was shown to induce apoptosis in RCC A498 and UOK121LN cell lines in a ER stress-, autophagy- and death receptor signaling-dependent manner [147]. More precisely, the combination of these two treatments led to the activation of the PERK branch and the subsequent inhibition of eIF2α, along with strong activation of the autophagic flux, eventually resulting in both apoptotic and non-apoptotic cell death events. The microtubule stabilizer Ixabepilone and the mTOR inhibitor Temsirolimus also led to significant induction of ER stress (GRP78 and CHOP increase) in renal cancer cell lines Caki-1 and Caki-2, triggering their growth arrest in vitro [148]. The combined effect of the histone deacetylase inhibitor Panobinostat and the human immunodeficiency virus protease inhibitor Nelfinavir were further tested in RCC [149]. This combination effectively induced ER stress (observed through GRP78 upregulation), histone acetylation and, to a great extent, cell death of RCC, both in vitro in 769-P, 786-O, Caki-2 RCC cells and in vivo in a xenograft mouse model of subcutaneous grafting of Caki-2 cells. Simultaneous treatments of Fluvastatin (statin-inhibiting cholesterol synthesis) and Vorinostat (histone deacetylase inhibitor) further resulted in decreased renal cancer growth in vitro in ACHN, A498 and Renca cells and in vivo in an allograft model of Renca subcutaneous injection in nude mice, through cooperative induction of histone acetylation and ER stress induction (detected as GRP78 upregulation) [150]. Finally, the HIV protease inhibitor Ritonavir, along with the proteasome inhibitor Delanzomib induced ER stress and inhibited the mTOR pathway, resulting in tumor growth arrest and suppressed colony formation in vitro in 769-P, 786-O, Caki-2 and Renca RCC cell lines and in in vivo mouse models of subcutaneous grafting of Renca cells [151]. A second study using Ritonavir—combined, this time, with Belinostat, a histone deacetylase inhibitor—led to a decrease in RCC growth and an induction of apoptosis, again, through ER stress activation [152]. It thus appears that combining different drugs that induce ER stress, such as histone deacetylases or microtubule destabilization agents, along with other drugs providing a second hit in cancer cells—such as proteasome inhibitors, which exacerbate the accumulation of misfolded proteins thus reinforcing ER stress—are promising therapeutic strategies for RCC.

Many different pharmacological ER stress modulators, e.g., synthetic or natural compounds, exist and usually target a specific branch of the UPR, as previously extensively reviewed [153]. Among those, only a handful were tested in the context of RCC and shown to trigger cell death. It remains, however, very difficult to quantify the relative extent of ER stress induced by these various compounds, since most of them were tested in different conditions and settings. For example, the plant extract englerin A was reported to induce a strong alteration of ceramide metabolism in RCC A498 cells in vitro, which, in turn, activated ER stress and acute inflammatory responses [154]. As RCC is a cancer type that is characterized by a strongly altered lipid metabolism, the authors suggest targeting this pathway in order to induce ER stress-mediated cell death. Another plant extract, withaferin A, was shown to induce ER stress in Caki RCC cells through the generation of ROS, thus triggering apoptosis of these cells [155]. Similar results were obtained in vitro in (i) RCC Caki cells treated with carnosic acid (rosemary extract); (ii) KCC853 cells treated with chitosan oligosaccharide (from the shells of shrimp and crab); (iii) Caki and 786-O cells treated with Chelerythrine (a protein kinase C inhibitor extracted from plants such as Chelidonium majus); (iv) 786-O, CaKi-1, ACHN and A-498 cells treated with norcantharidin (an anti-cancer drug inducing cell cycle arrest, isolated from natural blister beetles); and (v) A-498, 786-0 and ACHN cells treated with Bicyclol (a synthetic anti-hepatitis drug) [156,157,158,159,160]. The effect of RU486, a known progesterone and glucocorticoid receptor inhibitor, was also tested in RCC Caki cells, but in contrast to the previously mentioned compounds, which trigger ROS-dependent ER stress, this inhibitor induced CHOP and apoptosis through C/EBPδ-dependent mechanisms [161]. Finally, inflammatory cytokines such as Il-1β were reported to induce a strong ER stress sufficient to kill RCC cells. In the case of Il-1β, both in vitro and in vivo data using 786-O renal cancer cells xenografts in mice indicated that this cytokine induces dysregulation in protein folding accompanied by activation of monocyte chemoattractant protein 1 (MCP-1)/ MCPIP-1 signaling in RCC, which in turn activates ER stress (GRP78 and PERK increase) and ER stress-induced apoptosis (CHOP increase) [162].

Altogether, these studies demonstrated that ERST can be overcome by specific or combined stimuli in RCC cells, therefore supporting ER stress-mediated apoptosis in RCC as a relevant therapeutic approach. However, additional in vivo pre-clinical studies are required before envisaging such clinical applications, in order to better delineate the cellular and systemic effects of these ER stress inducers and to evaluate their potential relevance in clinics.

### 4.2. Nanoparticle-Mediated Hyperactivation of ER Stress in RCC

Nanoparticles made of silver, gold, copper, graphene and iron have recently been suggested as a strategy for cancer therapies, as these compounds have the ability to induce important cytotoxicity and to promote ER stress-mediated cell death [163]. Nanocatalyst-induced ER stress has also been tested for renal cancer therapy in an in vitro and in vivo setting (cultured RCC 786-O cells and a xenograft mouse model injected with 786-O cells) [164]. In this study, the administration of iron oxide nanoparticles (Fe_3_O_4_ NPs) leading to exacerbated ROS production, in particular through generation of ·OH by Fe_3_O_4_, damaged the ER in cancer cells and induced stress in this organelle [164]. Nevertheless, to avoid adaptive UPR responses in the RCC cell that would favor its survival, this strategy was coupled with the administration of a deubiquitinase inhibitor PR-619 treatment. PR-619 blocks the ERAD-dependent protein degradation axis, which is activated by UPR, and therefore contributes to a further exacerbation of abnormal protein accumulation and ER stress in RCC cells. This prolonged activation of ER stress led to apoptosis in cultured renal cancer 786-O cells and in the 786-O-derived tumors in a xenograft mouse model [164].

Another study reported the usage of cuprous oxide nanoparticles in order to disrupt normal copper transportation by altering the copper chaperone proteins ATOX1 and CCS in RCC cells [165]. A498 and SR786O RCC cell exposure to these nanoparticles triggered the accumulation of intracellular Ca^2+^ and ROS and subsequent ER stress, leading to the inhibition of migration and invasion, cell cycle arrest and apoptosis. In the same study, in vivo cuprous oxide nanoparticles administration led to the inhibition of tumor development in a RCC xenograft model of athymic BALB/c nude mice injected subcutaneously with 786-O or SR786O cells. Interestingly, these nanoparticles were also suggested to re-sensitize RCC cells to the TKI Sunitinib by reducing the expression of cellular factors promoting resistance to this TKI, such as AXL, MET, AKT, and ERK signaling effectors [165].

### 4.3. Inhibition of the UPR in Conjunction with TKI as a Therapy for RCC

**Figure 6:**
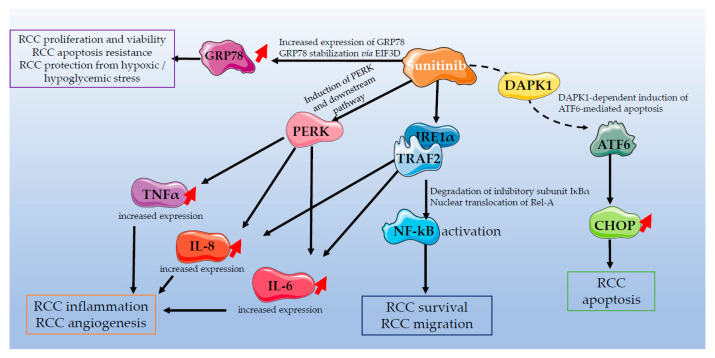
Interplay between Sunitinib treatment and ER stress signaling pathways in renal cell carcinoma. Graphical representation was designed using images from the Servier medical art database.

As previously mentioned, the TKI Sunitinib displays therapeutic effects only in a subset of RCC patients [43]. The inefficacy of currently used pharmacological therapies, in particular Sunitinib, was attributed to some extent to their ability to induce ER stress in a mild range and therefore to foster tumor survival instead of inducing apoptosis. It was indeed well demonstrated that Sunitinib activates ER stress in RCC (summarized in Figure 6) through different mechanisms. First, Sunitinib triggers the activity of pro-tumorigenic NF-kB, through the IRE1α/TRAF2/IKKβ signaling axis, therefore promoting cell survival, as reported in vitro in 786-O RCC cells [47]. In the same study, Sunitinib was shown to activate the PERK signaling branch of ER stress, leading to the production of inflammatory mediators such as Il-6, Il-8 and TNFα [47]. Similarly, treatment of the RCC Caki-1 cell line with Sunitinib in vitro increased the expression of GRP78, which can in turn lead to increased proliferation of the renal cancer cells in hypoxic/hypoglycemic stress situations and confer resistance to apoptosis by stimulating the PERK/eIF2α signaling axis [166]. The importance of GRP78 in Sunitinib resistance was also demonstrated in vivo in the same study, where xenografting of Caki-1 cells lacking the expression of GRP78 in nude mice resulted in significantly lower tumor growth, compared to Caki-1 cells expressing GRP78, when the mice were treated with Sunitinib [166]. Sunitinib was also shown to induce GRP78 indirectly in RCC by increasing the expression of the oncogene EIF3D, thus resulting in GRP78 stabilization and Sunitinib resistance, as observed in 786-O and ACHN cells [167]. Finally, ATF6, the third axis of ER stress, can also be stimulated by Sunitinib in 786-O and ACHN RCC cells with functional Death-Associated Protein Kinase 1 (DAPK1) expression [168].

As this mild, chronic activation of ER stress in RCC under TKI treatment confers resistance of RCC cells to death, targeting the UPR along with TKI treatment was considered in order to decrease their pro-survival and to restore their pro-apoptotic effects. This concept was particularly investigated for Sunitinib-based therapies. For example, downregulation of GRP78 expression by specific GRP78 siRNAs sensitized renal cancer Caki-1 cells to Sunitinib-induced apoptosis [169]. Similar results were described in RENCA renal carcinoma cells, where in vitro incubation with GRP78 siRNA lipoplex prior to exposure to Sunitinib triggered growth arrest [170]. As mentioned, Sunitinib was also described to activate the IRE1α and the PERK branches of the UPR in vitro in 786-O RCC cells, therefore increasing the expression of NF-kB, as well as those of the pro-inflammatory cytokines Il-6, Il-8 and TNFα [47]. The same study reported that inhibitors of PERK (GSK2656157) or IRE1α (4μ8C), or, alternatively, genetic deletion of PERK or IRE1α, significantly prevented overexpression of these inflammatory cytokines in 786-O RCC cells [47]. This study further allowed the conclusion that the different branches of the UPR signaling are not redundant, and, therefore, Sunitinib-mediated overexpression of NF-kB and RCC survival were mediated by IRE1α signaling, while the Sunitinib-mediated pro-tumorigenic cytokine increase was dependent of the PERK signaling [47]. A current clinical challenge consists now in understanding which branch of the UPR must be targeted along with Sunitinib treatment to minimize systemic toxicity while maximizing cell death of renal cancer cells.

### 4.4. Experimental Models to Investigate ER Stress in Renal Cell Carcinoma

Investigating the potential of targeting ER stress to treat RCC is challenging and hampered by the lack of highly relevant in vivo experimental models. The in vivo models most often used to study RCC include (i) xenograft models, which do not recapitulate cell transformation and tumor initiation in normal kidney tissues); (ii) genetic models of cancer induction in the kidney (e.g., knockouts of renal tumor suppressors in mice), which create a very specific setting of carcinogenesis poorly representative of the majority of RCC patients; and (iii) chemical induction of RCC, where the carcinogenic agents also affect other organs in the animal model, besides the kidney [171]. These important differences and drawbacks characterizing each type of RCC model challenge the establishment of relevant data on ER stress, which would reflect the pathophysiology in the vast majority of human RCC patients.

Because no currently available animal models faithfully recapitulate the human RCC pathologies and associated ER stress process, alternative experimental models need to be implemented to improve our understanding of the pathophysiological role of ER stress in RCC and the molecular mechanisms governing these processes. In this regard, studies with tumoral organoid cultures of RCC are encouraging. These tumoral organoids do indeed (i) retain more RCC characteristics than 2D cultures of cancer cells; (ii) have a human genome; (iii) can originate from different parts of the tumoral kidney; (iv) can contain different cell types; and (v) can be used to study ccRCC, pRCC or chRCC [172,173,174]. While the heterogeneity of tumoral organoids might appear to be a disadvantage in experimental settings, this variability illustrates the reality in patients and investigating a sufficient number of these tumoral organoids would likely bring relevant information about ER stress signaling in different types of RCC, on ERST in patients and on other relevant data about the impact of ER stress pharmacological modulators in tumoral progression. Similar studies performed recently to investigate dose responses of different TKI (Sunitinib, Sorafenib Axitinib, Pazopanib and Cabozantinib) on organoid viability further support the experimental use of such organoids to increase the relevance of these type of studies for human pathologies [175].

## 5. ER Stress as a Biomarker for RCC Prognosis

### 5.1. GRP78

A recent study highlighted GRP78 as a single prognostic marker in RCC involved in UPR signaling. GRP78 mRNA and protein levels were indeed increased in RCC, as well as serum levels, which correlated with the stage of the tumors [176]. GRP78 may thus represent an important potential non-invasive biomarker for RCC staging, but its relevance needs further confirmation in larger human patient cohorts.

Since RCC is frequently associated with ER stress, an increase in cellular and serum levels of GRP78 may seem counterintuitive in RCC, since GRP78 is a gatekeeper of UPR activation that binds and restrains the activity of IRE1α, PERK and ATF6. Actually, the function of GRP78 is more complex than just inhibiting the UPR in the ER. Alternative splicing of GRP78 was shown to target the protein to different cellular compartments, such as the cytoplasm, the mitochondria, the nucleus and the cell surface [177]. For example, the cytosolic isoform of GRP78 results from an alternative splicing event involving retention of the first intron and subsequent internal translation initiation, finally leading to the loss of the ER-targeting signal [178]. Non-canonical functions of GRP78 were further reported to associate with its localization outside of the ER, such as (i) inhibition of DNA damage-induced apoptosis in the nucleus or (ii) regulation of cell survival signaling pathways such as the PI3K/AKT signaling at the cell surface [177]. So far, these non-canonical functions of GRP78 outside of the ER have not been investigated specifically in RCC but could potentially promote RCC survival. Finally, it is also possible that increased levels of the canonical ER-lumen form of GRP78 in RCC cells prevent ER stress hyperactivation and maintain UPR activation under the ERST. This would allow RCC cells to avoid pro-apoptotic signaling under mild ER stress while still maintaining the benefits of weak UPR activation. Future studies should shed light on the potential role of GRP78 in determining the ERST in RCC.

### 5.2. ER Stress Gene Signature

**Figure 7:**
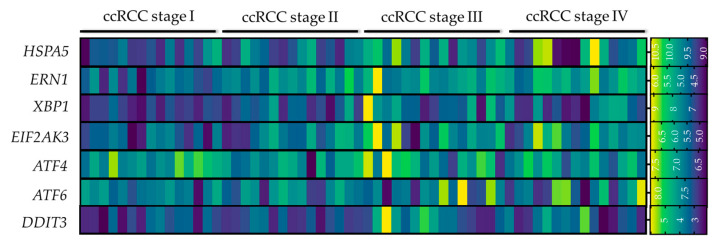
Expression of ER stress mediators in human ccRCC biopsies segregated in different stages. Heatmap representation of the expression of *HSPA5* (encoding GRP78), *ERN1* (encoding IRE1α), *XBP1*, *EIF2AK3* (encoding PERK), *ATF4*, *ATF6* and *DDIT3* (encoding CHOP) in human ccRCC biopsies at stage I, II, III and IV (*n* = 15/group). The data were acquired from the GEOdataset GSE150404 (https://www.ncbi.nlm.nih.gov/geo/query/acc.cgi?acc=GSE150404 accessed on 26 December 2022–expression profiling by microarray) and were analyzed via the GEO2R software. Graphical representation was designed with the GraphPad Prism 9 software.

Retrospective in silico analyses of RCC patient datasets have allowed for the identification of ER stress signatures tightly correlated with the outcome of the patient. Indeed, consensus-clustering of the ccRCC patients from the TCGA cohort depending on their ER stress-related gene expression led to the formation of two different clusters (C1 and C2). This segregation of the patients in two clusters highlighted that the ER stress signature can vary greatly between patients. Strikingly, the clinical features of the tumors in each ER stress cluster were different, with RCC samples belonging to the C2 being more advanced/aggressive (more advanced tumor T stage, TNM stage and grade level) in comparison to C1 RCC [179]. Interestingly, the C2 cluster displayed a significant increase in PERK and ATF6 expression in contrast to C1, whereas IRE1α expression remained unchanged. Coincidentally, patients clustered in the C2 were better responders to Sunitinib compared to patients in C1, potentially because of their higher basal activation of the UPR. The extent of ER stress signaling based on this gene signature was further correlated with the type of immune responses in the patients’ tumors [179]. Indeed, the infiltration ratios of regulatory T (T regs) and CD8 T cells were higher in the C2, compared to the C1 cluster, whereas infiltration levels of monocytes, neutrophils, M1 macrophages, dendritic cells and mast cells were higher in C1 cluster. These correlative observations need to be further confirmed by more extensive clinical data, however. Based on these in silico analyses, an ER stress-related prognostic risk model has been established for RCC, suggesting ER stress signatures be considered not only for patient prognosis but also for the design of appropriated therapeutic strategies [179]. A similar in silico study of the TCGA cohort confirmed these findings by identifying an 8 ER stress-related gene prognostics signature that could be used to determine whether the outcome of the patient is high- or low-risk (in terms of prognosis), with high-risk patients presenting more important immune infiltration and higher immune scoring [180]. Our own analyses of the publicly available GEO dataset GSE150404 allowed us to also observe that more advanced stages of human ccRCC (stage III and IV) are associated with higher expression of UPR mediators, compared to early ccRCC stages (Figure 7).

## 6. Conclusions

RCC is a global health issue, due to poor patient survival for this cancer and the low efficiency of treatments currently available [181]. Indeed, while nephrectomy and radio-ablation, as well as first line treatments such as TKI, immunotherapies and combinatory strategies remain valid therapeutic options, the criteria of eligibility for these treatments are restrictive and the efficiency of these therapeutic approaches is patient-specific. When considering the therapeutic targeting of ER stress, two different approaches can be envisaged based on currently available data. First, ER stress can be pharmacologically hyperactivated to a point overpassing the ERST, where damage caused by the ER stress induction is deemed irreversible in RCC cells and therefore leads to cell death. Second, pharmacological inhibition of a specific branch of the UPR, or global suppression of UPR signaling, could be performed to prevent mild ER stress induction of pro-survival mechanisms in RCC cells, along with another drug, such as TKI, to induce death of cancer cells.

Chronic ER stress activation is a key hallmark of RCC that helps cancer cells cope with hypoxia, lack of vascularization in early-stage tumors and scarce nutritional conditions, e.g., by activating autophagy in order to efficiently grow and proliferate. The exact mechanism by which cancer cells manage to survive and not undergo apoptosis during chronic UPR activation remains poorly understood but could result from selective attenuation of specific UPR signaling, as well as epigenetic or post-translational negative regulation of ER stress mediators, as has been suggested for CHOP [182].

The threshold of ER stress tolerance shifting this process from an anti-apoptotic to a pro-apoptotic event and whether this threshold is dependent of a specific branch of the UPR, such as the PERK or ATF6 induction of CHOP, also currently remain poorly understood. Moreover, how patient genetic specificities, lifestyle and the type or mutations in RCC determine ERST is also unknown but deserves in depth investigation prior to clinical application of ER stress-targeting therapies for RCC treatment. Finally, key master regulators, e.g., E2F1 [58], that potentially switch the UPR from an adaptive mechanism to s pro-apoptotic process when ERST is attained need to be further identified and characterized. As well, the weight of indirect mechanisms that can also impact ERST with chronic ER stress, e.g., the PERK-mediated inhibition of protein translation [183] or prolonged IRE1α endonuclease activity [183], are important questions that need extensive clarification before considering targeting of the UPR for therapeutic purposes.

Finally, the decision to effectively hyperactivate ER stress in RCC or to sensitize RCC to specific treatment by suppressing ER stress signaling needs to include the determination and standardization of basal UPR activation levels in patients’ tumors in order to proceed with such personalized medicine approaches. New standardized procedures and quantitative measures of ER stress in patients’ tumors needs to be developed in addition to the classical analyses of ATF4/GRP78 mRNA expression, XBP1 splicing or protein analyses of total of phosphorylated UPR effectors (IRE1α, eIF2α, cleaved and total ATF6, total PERK, CHOP and GRP78) [184]. This requires establishing a standardized multi-parametric activation/inhibition range for the UPR that would clearly indicate the strategy of ER stress modulation that would be best for a given patient.

## Data Availability

Not applicable.
